# Statistical Inference on the Mechanisms of Genome Evolution

**DOI:** 10.1371/journal.pgen.1001389

**Published:** 2011-06-09

**Authors:** Michael Lynch

**Affiliations:** Department of Biology, Indiana University, Bloomington, Indiana, United States of America; Yale University, United States of America

## Introduction

In a series of publications, I and my colleagues have developed hypotheses for how the evolution of various aspects of genome architecture is expected to proceed under conditions in which the forces of random genetic drift and mutation predominate (e.g., [1]–[15]). These models, collectively referred to below as the mutational-hazard (hereafter, MH) hypothesis, are sometimes represented as neutral models [16], [17], but this is not correct, as the key component of each model is the deleterious mutational consequence of excess DNA. The MH hypothesis is, however, a nonadaptational model, in that it yields expectations on the structure of genomes without invoking external selective forces.

It is likely that some aspects of these models will need to be changed as more is learned about the molecular consequences of various aspects of gene structure and the nature of mutation. Such modifications will not alter the need for baseline null hypotheses in attempts to defend adaptive explanations for variation in genomic architecture [9]. Nevertheless, any theory that strives to provide a unifying explanation for diverse sets of genomic observations must be scrutinized extensively from a variety of angles and interpreted in the context of well-established molecular and population-genetic processes. Although I will argue that a recent challenge to the MH hypothesis by Whitney and Garland ([18]; hereafter, WG) contains numerous problems, this exchange may help clarify more broadly misunderstood issues.

## Errors in Statistical Logic and Analysis

Statistical theory provides a framework for rigorously testing hypotheses in biology, with two of the more dramatic examples being the formal theory of quantitative genetics [19] and phylogenetic inference [20]. Nevertheless, the utility of statistical methods for hypothesis testing depends critically on the extent to which the underlying model assumptions match the features of the system under investigation. Like an ill-defined verbal argument, overconfidence in an inappropriate quantitative analysis can lead to misleading interpretations.

Unfortunately, because large-scale changes in genomic architecture emerge on time scales of tens to hundreds of millions of years, tests of general theories of genome evolution are highly reliant on comparative data. This can raise issues regarding the significance of hypothesis tests when the underlying data share evolutionary history. Since Felsenstein [21] introduced the rationale for the phylogenetic comparative method, various derivative techniques have been developed, some by the author of this paper [22], [23]. These approaches have been used broadly in evolutionary ecology, although not always with good justification (as emphasized in [24]–[26]). Using such methods, WG concluded that phylogenetic diversity of genomic features is unaffected by variation in the power of random genetic drift, challenging the MH hypothesis, but there are at least four classes of statistical problems associated with this study.

First, the analyses employed by WG are only justified when the characters under consideration have some possibility of shared evolutionary history among related taxa. The degree to which history is shared across related lineages is often unclear with phenotypic traits. However, the issues are well-understood for the central variable in the analyses of WG, the level of average nucleotide heterozygosity at silent sites (π_s_), which has an expected value of *N*_e_ *u* under mutation-drift equilibrium (where *N*_e_ is the effective population size, and *u* is the base-substitutional mutation rate per nucleotide site; ignoring, for simplification, the factor of 4 or 2 that should precede this expression in diploid versus haploid populations).

The expected coalescence time for a neutral gene genealogy, 4*N*_e_ generations in a diploid species, is dramatically less than the divergence time between even the most closely related species in WG's analysis (e.g., *Mus* and *Homo*, *Drosophila* and *Anopheles*, none of which share ancestral polymorphisms). Therefore, if any trait can be stated as having no shared phylogenetic history in the analyses of WG, it is the *estimator* of *N*_e_ *u*. Although all traces of ancestral π_s_ values have been erased many times over for the taxa in this study, one could perhaps still argue that some shared history remains with respect to the underlying population size and mutation rate determinants in some pairs of lineages, which might allow similar heterozygosity values to re-emerge. It is notable, however, that there is considerable turnover among lineages in the genes encoding for enzymes that dictate the mutation rate, with the replication polymerases in eukaryotes and eubacteria not even being orthologous, and the repair polymerases in numerous eukaryotic lineages being absent from others. In any event, this concern is dwarfed by other limitations, including the very high sampling variance associated with π_s_ estimates (the standard errors of estimates often being of the same order of magnitude as the estimates themselves), and the unknown element of temporal variation on time scales exceeding *N*_e_ generations. Because of such enormous sampling variation, this author has generally simply reported average estimates of π_s_ across wide phylogenetic groups (e.g., [5]). By deriving independent contrasts on π_s_, WG greatly inflated the sampling variance of this parameter, and it can be shown that this problem alone will cause a ∼30% decline in expected *r* ^2^ values involving correlations with other traits.

An equally substantial problem is associated with the strict interpretation of π_s_ as a measure (or linear correlate) of *N*_e_ *u* across all of life. Most notably, many prokaryotes appear to approach the maximum level of *N*_e_ (and minimum level of *u*) dictated by the effects of selection on linked genes [7], [15], in which case, the independent contrasts of true values of *N*_e_ *u* between such species pairs will be essentially randomly distributed around zero. This problem is compounded by the downward bias in π_s_-based estimates of *N*_e_ *u* in unicellular species that results from selection on silent sites [5], [7], [27], [28]. Even if we can be confident that *N*_e_ *u* is much higher in prokaryotes than in vertebrates, the estimates based on π_s_ may be off by more than an order of magnitude [7].

Owing to the long time scale on which genomic alterations accrue, the concern for shared evolutionary history in such attributes might in some cases be more justified. However, for the lineages evaluated by WG, such phylogenetic inertia is overshadowed by other evolutionary effects. For example, for the two most closely related species included in the WG analysis, mouse and human (and most other eutherian mammals), numerous shared features of genome architecture are a consequence of convergent evolution, not shared ancestry [29]; the same is true of the ancestral species leading to the land-plant and metazoan lineages [7]. The complete turnover of various mobile-element families among eukaryotic lineages provides additional compelling evidence for the absence of strong phylogenetic effects among the taxa examined by WG. Thus, as in the case of factors influencing the mutation rate, it is unclear whether the aspects of shared biological history that are the targets of the WG analysis are any more meaningful than applying a similar strategy in combined study of bat, bird, and insect wings.

Second, use of a phylogenetic tree with questionable branch lengths will further obfuscate any phylogenetic analysis, as branch-length scaling must yield uniform sampling variances of the contrast data for downstream hypothesis tests to be valid. In an attempt to remove such issues, WG standardized all branch lengths to unit length, although there are no obvious evolutionary models that would produce the desired behavior for the characters examined. The relevant time scale for evolutionary processes is the number of generations per branch, whereas phylogenetic trees are simply based on net accumulations of nucleotide substitutions. Under the assumption that the molecular sites on which a tree is based are neutral (which can be questioned), the rate of mutation accumulation would be proportional to the product of the per-generation mutation rate and the number of generations elapsed. The first quantity varies by approximately two orders of magnitude among the species in this study [15], and the generation length varies by more than five orders of magnitude (from <1 hour to ∼20 years). Thus, at the very least, the consequences of the arbitrary scaling to equal branch lengths are obscure.

A more significant issue is the validity of the topology of the phylogenetic tree employed. WG appear to have simply spliced together subtrees from several independent studies, many aspects of which continue to be highly debated. These include the issues of whether echinoderms and tunicates are monophyletic, and whether nematodes and arthropods are united in the ecdysozoa. Most phylogeneticists agree that the deep branching positions of all of the major eukaryotic lineages other than animals, fungi, and slime molds are highly uncertain. Thus, although some phylogenetic nonindependence may have been removed in the analyses of WG, numerous spurious internal relationships were also likely created, rendering the analysis much less rigorous than the authors imply.

Third, perhaps the most fundamental issue of the analysis of WG is the very nature of the hypothesis test that was carried out. Although the authors assumed that various measures of genome architecture will be linearly related to π_s_ on a logarithmic scale under the MH hypothesis, this is not what the theory predicts. Rather, the theory predicts a threshold response to *N*_e_ *u* (or *N*_e_) for many aspects of genome architecture, and such scaling can be seen in many genomic contexts, ranging from intron investment to mobile-element contributions to genome size itself [7]. Failure to account for this feature naturally eliminates any obvious scaling with *N*_e_ *u* when independent contrasts are employed. For example, if most pairs of species reside to the right or left of a threshold, which is certainly the case with the taxa examined by WG, an independent-contrast analysis will produce a situation in which nearly all contrasts have expected values equal to zero, yielding a near-zero correlation (and removing all positional information with respect to the threshold). Thus, rather than being a contradiction of the MH hypothesis, a substantial reduction in the correlation of genomic attributes with the independent contrasts of π_s_ employed by WG is completely consistent with theoretical expectations.

Finally, it should be noted that when the features of the underlying data do not violate the assumptions of a statistical model (which is not the case in the WG study), ordinary least-squares correlations are, on average, unbiased with respect to the true underlying parameter, i.e., species sampling simply leads to greater noise among individual samples, but does not alter the average outcome [23], [26], [30]. Consequently, unlike the aberrant behavior observed by WG, relationships that evolve in a double-diffusion-like process generally yield similar correlations whether or not shared phylogenetic history is accounted for [24].

To improve the quality of future work in comparative genomics, WG advocate an even broader use of phylogenetic methods. However, unless a model more relevant to the tempo and time scale of evolution of the components of genomic evolution is incorporated, unless unbiased estimators of *N*_e_ *u* can be procured, and unless appropriate metrics and topologies of the underlying phylogenies can be obtained, it appears that the methods being promoted by WG will be no more informative than ordinary least squares and may even continue to be misleading.

## Biological Misinterpretations

To strengthen their argument that drift has little influence on genome architecture, WG claim that three other sets of observations are inconsistent with the MH hypothesis. For example, they note that Whitney et al. [31] found a low correlation of genome size with estimates of *N*_e_ derived from measures of allozyme heterozygosity in a wide variety of plants. Contrary to the authors' arguments, such estimates of *N*_e_ are quite problematic. First, because allozymes are functions of protein-sequence variation, they are much less reliable surrogates of neutral variation than silent sites. There is no theoretical basis for a positive correlation between allozyme variation and *N*_e_ *u*, and if there is substantial selection on allozymes, the relationship could even be negative. Second, although the authors extrapolated estimates of *N*_e_ by dividing levels of allozyme heterozygosity by a mutation rate of *u* = 10^5^ per allele per generation (the basis of which is unclear), even if the assumption of neutrality were correct, this is an inappropriate manipulation. Per-generation mutation rates vary substantially across species in such a way that the very strong negative correlation between *N*_e_ and *u* results in π_s_ scaling only weakly with *N*_e_ [15]. Thus, although the observations in [31] are again superficially consistent with the MH hypothesis, no confident conclusions can be drawn from the results.

WG also suggest that the tendency for microbial genome sizes to decline with decreasing *N*_e_ [32] is inconsistent with the MH hypothesis. In fact, the opposite is true—the theory predicts that with increasing power of random genetic drift, effectively neutral genomic features will diverge in the direction of mutation bias. Because there is a deletion bias in bacteria, the observation of Kuo et al. [32] actually provides compelling support for the MH hypothesis, in that a pattern different from that in eukaryotes (where there is an insertion bias due to a strong contribution from mobile-element insertions) is both predicted and observed. Notably, this shift in the direction of mutation pressure is also a striking violation of the underlying assumption of a constant background pattern of stochastic evolution in the linear independent-contrasts methods employed by WG.

In advocating the need for better estimators for *N*_e_, WG emphasize the utility of the *K*_a_/*K*_s_ ratio of nonsynonomous to synonymous divergence, which is often used as a measure of the efficiency of selection. However, this overlooks two significant issues. First, the theoretical expectations of the MH hypothesis are not a simple function of *N*_e_ but of the product *N*_e_ *u*, which is the ratio of the power of mutation to the power of drift. Thus, the criticism that an estimator of *N*_e_ *u* is a poor proxy for *N*_e_ is misplaced, as it is the former that is critical to testing the MH hypothesis, whereas the latter is insufficient. Fortunately, it is easier (although, as noted above, not easy) to estimate *N*_e_ *u* than *N*_e_. Second, the *K*_a_/*K*_s_ index at best provides an estimate of the average efficiency of selection operating on amino acid substitutions, whereas the MH hypothesis is focused on the vulnerability of gene/genome-structural modifications to mutation pressure. There is no theoretical or empirical basis for expecting *K*_a_/*K*_s_ to covary with *N*_e_ *u*. Although commonly used, it is not even clear that *K*_a_/*K*_s_ scales appropriately with the efficiency of selection in populations of large size. If, for example, *N*_e_ is sufficiently large that nearly all nonsynonomous changes involve neutral substitutions, any further increase in *N*_e_ will have no effect on *K*_a_ while reducing *K*_s_, and hence reducing *K*_a_/*K*_s_ (contrary to the assumption that low *K*_a_/*K*_s_ implies large *N*_e_).

## Moving Forward

In questioning the role of drift, and apparently mutation (based on their treatment of it as a nuisance parameter), in the evolution of genomic attributes, WG provide no alternative explanations for the numerous patterns of genomic structural variation known to exist within and among prokaryotes and eukaryotes. In contrast, the MH hypothesis provides a potential solution to the problem of why various aspects of animal and plant genomes evolve in opposite directions within organelles while converging within the nucleus; that the explanation is related to variation in *u* rather than *N*_e_ further demonstrates the difficulty of focusing solely on *N_e_*, in accordance with the dual nature of the proposed process [13]. The MH hypothesis provides a plausible explanation for the expansion but near constancy of average UTR lengths in eukaryotes [12], for various aspects of intron evolution [4], [33], and for numerous features in nonrecombining chromosomal regions [7]. The model expectations are also consistent with the genomic modifications incurred by endosymbiotic bacteria, and with the remarkable convergence of the features of integrated polydnaviral genomes on those of their insect host chromosomes. Finally, the hypothesis provides an explanation for the parallel contraction in numbers of retrotransposons, pseudogenes, and insertions of mitochondrial DNA into the nuclear genomes of independent mammalian lineages following the post-KT geographic expansion of mammals [29]. In short, the evidence that excess DNA is associated with weak mutational disadvantages is compelling, and by invoking the inability of selection to oppose such changes in populations of sufficiently small size, the MH hypothesis provides a potentially unifying explanation for a diversity of previously disconnected observations.

Given its broad phylogenetic perspective across species with widely different features, the MH hypothesis is admittedly difficult to test with comparative data. However, the general theory is based on fundamental principles of population genetics that transcend species boundaries and are readily evaluated with modern-day organisms. For example, the deleterious nature of introns has recently been demonstrated in at least two ways (e.g., [33], [34]), and suggestions have been made as to how models on duplicate-gene evolution might be tested with information on within-species polymorphisms [35]. Nonetheless, legitimate questions about the breadth of applicability of the theory remain to be answered [36], [37]. The hypothesis cannot explain the precise gene content of species, which must be molded to a large extent by the environment. Nor can it explain all aspects of “noncoding DNA,” as some of this territory has positive functions. Additional complications arise from the fact that some modern-day genomes have structures that are out of equilibrium with current effective population sizes (e.g., [29]), a factor that may explain the apparently complex genome of the ancestral eukaryote and the continuing loss of such complexity in many of today's unicellular lineages [7], [38], [39].

Future observations on key phylogenetic lineages varying in significant ways with respect to long-term intensities of mutation, drift, and recombination will provide the observations on which the MH hypothesis will stand or fall. Improvements are already possible, now that mutation rates can be directly measured in a wide variety of genomes with high-throughput sequencing [15]. Unfortunately, the procurement of direct estimates of *N*_e_ remains dauntingly difficult [40], and until this problem is solved, it will remain difficult to obtain unbiased estimates of the key parameter *N*_e_ *u.* However, there is no justification for rejecting a theory based on its accessibility to formal hypothesis testing. It can be tempting to invoke observations on single genomes as being in support or conflict with the MH hypothesis [41]–[44], but due to the stochastic nature of evolutionary processes, the full domain of applicability of the model will only be known after the accumulation of many such observations. Well-reasoned applications of statistics will surely play a role, but the real advances will come from an enhanced understanding of genome biology.
